# Supplementary material for: Divergent dynamics of sexual and habitat isolation at the transition between stick insect populations and species
Source: Nat Commun. 2024 Mar 13;15:2273. doi: 10.1038/s41467-024-46294-9 (PMC10937975; doi:10.1038/s41467-024-46294-9)
Supplement: Supplementary file 1 — Supplementary Information [file 41467_2024_46294_MOESM1_ESM.pdf]

Supplementary Information for: Divergent dynamics of sexual and habitat isolation at the transition between stick insect populations and species

Patrik Nosil[1], **Zachariah Gompert**[2], **Daniel J. Funk**[*,3]

[1] CEFE, Univ Montpellier, CNRS, EPHE, IRD, Montpellier, France

[2] Department of Biology, Utah State University, Logan, UT, USA

[3] Department of Biological Sciences, Vanderbilt University, Nashville, TN, USA

**author to whom correspondence should be addressed*
daniel.j.funk@vanderbilt.edu

Supplementary Methods

Inferences of Divergence Time and Gene Flow. Gene flow and other demographic events, combined with the numerical properties of genetic distance measures, can influence the relationship between time and genetic distance. Thus, as described in the main text, we used a combination of phylogenetics and historical demographic inference to quantify the relationship between divergence time and genetic distance (F_{ST}) in *Timema* with the aim of better understanding the processes affecting the observed patterns with respect to genetic distance. We first estimated divergence times between population pairs using a time-calibrated, population-level phylogeny [1]. For this, we focused on pairs of populations unlikely to experience gene flow at present, that is inter-specific pairs and allopatric conspecific populations (Supplementary Table 3). Past work has shown a lack of gene flow between *Timema* species [1, 2], and little to no gene flow beyond about 15 km for *T. cristinae* populations [3]. We extracted divergence time point estimates (median of the posterior distribution) from the published Bayesian phylogenetic analysis [1] (Supplementary Table 3). We then fit and compared regression models for genome-average F_{ST} as a function of divergence time. This was done using the `lm` and `glm` functions in `R`. We found a strong but non-linear relationship between divergence time and F_{ST} (Supplementary Table 3, Fig. 7A). Specifically, a quadratic polynomial model outperformed a simple linear model (Akaike Information Criterion [AIC] of -14.94 versus -9.728, respectively) and explained 78% of the variation in F_{ST} ($N = 14$ taxon pairs, $P = 0.0002$); this model suggests F_{ST} accumulates more slowly with time after about 10 million years of divergence.

We complemented this phylogenetic approach by estimating divergence times between allopatric and sympatric/paraptric pairs of conspecific populations under an isolation-with-migration (IM) model using the diffusion approximation implemented in $\delta a \delta i$ [4, 5]. To do this, we re-analyzed the published GBS data from 10 intraspecific population pairs, including *T. podura*, *T. poppensis*, *T. knulli*, *T. curi*, and *T. chumash* (Supplementary Table 4) [1]. This includes all conspecific pairs from [1] that were also part of the current study, with the exception of *T. cristinae* pair 6, which was excluded because the sample size was low. Past work with these data required a common (shared) set of SNPs across species and was based on aligning all of these data to an earlier draft of the *T. cristinae* genome [1]. Here, we were interested in only conspecific comparisons, that is, pairs of populations where gene flow might be recent or ongoing. Because of our focus on conspsecific pairs, we did not require a common set of SNPs across all species (just for populations within species). Thus, we aligned the sequence data from each species to the closest, chromosome-scale genome assembly: the *T. chumash* genome [6] for *T. chumash* and *T. podura*, and the *T. knulli* genome (NCBI Genome SUB13282982) for *T. knulli*, *T. poppensis* and *T. curi*. We did this using the `mem` algorithm from `bwa` (0.7.17-r1198-dirty) [7]. For alignment, we set the minimum seed length to 19 bps and looked for internal seeds within seeds longer than 19 × 1.5 bps. We then compressed, sorted and indexed the alignments with `samtools` (version 1.16) [8]. Next, we separately identified SNPs within each species using `bcftools mpileup` and `bcftools call` (version 1.16) [8, 9]. For variant calling, we skipped reads with quality scores less than 20 and bases with quality scores less than 30. We also ignored putative insertion/deletion polymorphisms. We used the classic (consensus) variant caller (`-c`) and only output SNPs if the posterior probability that the site was invariant was < 0.01. We then filtered the resulting vcf file for each species to retain only SNPs with ≥2X coverage per individual, z-scores < 3 for base-quality bias, mapping-quality bias and read-position bias tests, quality scores ≥ 30 and missing data (zero reads covering a SNP locus) in fewer than 20% of individuals. This left us with 17,574 SNPs for *T. chumash* (N = 358 individuals), 14,433 SNPs for *T. podura* (N = 244 individuals), 27,289 SNPs for *T. curi* (N = 85 individuals), 78,404 SNPs for *T. knulli* (N = 76 individuals), and 35,010 SNPs for *T. poppensis* (N = 116 individuals). The filtered vcf files were used to create joint site-frequency-spectra for demographic inference within $\delta a \delta i$ [5].

We then fit isolation-with-migration (IM) models for each of the 10 population pairs using $\delta a \delta i$. This involved estimating the following parameters: an ancestral effective population size ($\theta = 4N_e\mu$), split proportion (s), the split time ($T_{split} = 2N_e$ generations), population growth parameters (ν_1 and ν_2, size of each population relative to the ancestral population), and migration rates M_{12} and M_{21} (number of migrants assuming the ancestral effective population size, N_e). The `python` specification of the IM model was as follows:

```
def IM(params, ns, pts):
```

```
        s, nu1, nu2, T, m12, m21 = params
        xx = Numerics.default_grid(pts)
        phi = PhiManip.phi_1D(xx)
        phi = PhiManip.phi_1D_to_2D(xx, phi)
        nu1_func = lambda t : s * (nu1/s) ** (t/T)
        nu2_func = lambda t : (1-s) * (nu2/(1-s)) ** (t/T)
        phi = Integration.two_pops(phi, xx, T, nu1_func,
              nu2_func, m12 = m12, m21 = m21)
        fs = Spectrum.from_phi(phi, ns, (xx, xx))
        return fs
```

Prior to analysis, we down-sampled the data to 80% of the original sample sizes to minimize the effects of missing data. Grid points for extrapolation were set to $\bar{N}$, $\bar{N} + 10$ and $\bar{N} + 20$, where $\bar{N}$ is the mean sample size for a population pair (in gene copies, and after down-sampling). We considered the following value ranges for numerical optimization of parameters: 0.1 to 0.9 for s, 0.1 to 10 for ν_1 and ν_2, 0 to 10 for T_{split}, and 0 to 8 for M_{12} and M_{21}. We obtained initial values by applying a small perturbation to $s = 0.5$, $\nu_1 = 0.8$, $\nu_2 = 1.2$, $T_{split} = 0.4$, $M_{12} = 0.5$, and $M_{21} = 0.5$. We set θ to the optimal value conditional on the other model parameters. We then conducted multiple rounds of numerical optimization to obtain the maximum likelihood parameter estimates. The first step involved 20 rounds of optimization, each comprising 20 iterations. Each round began from a perturbation of the parameter estimates from the previous round. An additional 10 rounds of optimization were then performed, initially starting from a perturbation of the best parameter estimates from the first 20 rounds. These 10 rounds of optimization each comprised 30 iterations. A final five rounds of optimization were then performed starting from a perturbation of the best estimates from the previous 10 rounds. Each of these final five rounds involved 50 iterations for optimization. The best parameter estimates (i.e., the parameter values with the highest likelihood) were taken as the final maximum likelihood estimates. If optimization failed to converge (i.e., if the optimization procedure failed to finish), we repeated the entire process after sub-sampling to 50% of the original data.

The divergence time estimate for one of the ten pairs, *T. podura* BMTC versus DZRA (pair 13), was about an order of magnitude greater than the estimates for the other pairs (Supplementary Data 5). Excluding this outlier pair, these estimates of divergence time were strongly related to F_{ST} (Fig. 7B and Supplementary Fig. 2B). Quadratic polynomial models for F_{ST} were preferred by AIC when considering time in natural coalescent units (i.e., $2N_e$ generations) or relative to mutation rate (obtained by dividing time estimates by $\theta = 4N_e\mu$), and these models explained 93.7% ($N = 9$ population pairs, $P = 0.0002$) or 78.6% ($N = 9$ population pairs, $P = 0.0098$) of the variation in F_{ST}, respectively. An asmyptote was evident for time relative to mutation rate but not time in coalescent units (Fig. 7B and Supplementary Fig. 2B).

We further compared this isolation-with-migration model to two alternatives, a strict isolation (SI) model and a model of isolation followed by secondary contact (SC). Specifications for these models were as follows:

```
def SI(params, ns, pts):
        s, nu1, nu2, T = params
        xx = Numerics.default_grid(pts)
        phi = PhiManip.phi_1D(xx)
        phi = PhiManip.phi_1D_to_2D(xx, phi)
        nu1_func = lambda t : s * (nu1/s) ** (t/T)
        nu2_func = lambda t : (1-s) * (nu2/(1-s)) ** (t/T)
        phi = Integration.two_pops(phi, xx, T, nu1_func, nu2_func, m12 = 0, m21 = 0)
        fs = Spectrum.from_phi(phi, ns, (xx, xx))
        return fs

def SC(params, ns, pts):
    s, nu1, nu2, Ts, Tsc, m12, m21 = params
    xx = Numerics.default_grid(pts)
    phi = PhiManip.phi_1D(xx)
```

```
    phi = PhiManip.phi_1D_to_2D(xx, phi)
    nu1_func = lambda t : s * (nu1/s) ** (t/Ts)
    nu2_func = lambda t : (1-s) * (nu2/(1-s)) ** (t/Ts)
    ## split with no gene flow
    phi = Integration.two_pops(phi, xx, Ts, nu1_func, nu2_func, m12 = 0, m21 = 0)
    ## secondary contact
    phi = Integration.two_pops(phi, xx, Tsc, nu1_func, nu2_func, m12 = m12, m21 = m21)
    fs = Spectrum.from_phi(phi, ns, (xx, xx))
    return fs
```

The strict isolation model was identical to the IM model, except the migration rate parameters were fixed at 0. The secondary contact model assumed an initial period of strict isolation (lasting for time T), followed by secondary contact at T_{sc} generations in the past. Thus, the divergence time under this model (T_{split}) was $T + T_{sc}$. Bounds and optimization procedures for these models followed those for the IM model, except for these specific differences in parameter bounds: $T = 0$ to 10 (initial value = 0.4) and $T_{sc} = 0$ to 5 (initial value = 0.2). If any of the models failed to converge with 80% sub-sampling, all three models (including the IM model) were re-fit with 50% sub-sampling. We examined the overall fit of the best model by comparing observed and predicted site-frequency-spectra for each population pair to ensure the best model was a reasonable fit for the data before proceeding.

The best model based on AIC varied across population pairs, with each model being preferred for at least one pair (the secondary contact model failed to converge for pair 18) (Supplementary Data 5 and Supplementary Table 4). When considering the best model for each pair, we failed to detect a significant relationship between divergence time and F_{ST}, though the trend was consistent with the results from the isolation-with-migration models ($N = 10$ population pairs, quadratic polynomial model, $P = 0.2027$ and 0.1080 for coalescent time and time relative to mutation, respectively).

Bayesian Model for Sexual Isolation with Uncertainty. In the main text, we presented results from fitting Bayesian linear mixed models for sexual isolation as a function of genetic distance, taxonomic status and the interaction between these two variables. These models did not explicitly account for uncertainty in the sexual isolation index, although such uncertainty should be mostly captured by the residual (error) variance. We thus fit an additional set of models (the full model and all more restricted sub-models, as described in the main text) that fully and explicitly incorporated uncertainty in the sexual isolation index. For this, we modified the model described in the main text as follows. We assumed that the true, unknown, sexual isolation index followed a normal distribution with a mean given by the linear model and a residual error (parameterized by a precision parameter, τ_e). We further assumed that the measured sexual isolation index followed a normal distribution with a mean given by the true, unknown, index and a variance, again parameterized as a precision, τ_m. This variance was estimated from the data prior to the Bayesian analysis. Specifically, we fit Bayesian beta-binomial models for the observed counts of success for each mating type for each taxon pair (binomial likelihood) with an mostly uninformative beta prior ($\alpha = 1$, $\beta = 1$). The resulting beta posterior, which can be computed analytically, was then sampled 1000 times (in `R` with the `rbeta` function), to obtain expected variances for the sexual isolation index (which is a function of the proportion of each of four mating pairs that was successful). In all other ways, this model was fit (again using `rjags` with `R` version 4.2.2) in precisely the same manner as the model describe din the main text.

Here, the best model was again the model with only genetic distance (DIC = 37.84), whereas the worst models were the null model (DIC = 42.91) and the model with only taxonomic status (DIC = 42.52). Under the model with only genetic distance (the best model), there was an positive relationship between genetic distance and sexual isolation ($\beta = 0.31$, 95% ETPI = 0.12 to 0.51, posterior probability $\beta > 0 \approx 1$).

References

[1] Riesch, R. *et al.* Transitions between phases of genomic differentiation during stick-insect speciation. *Nature Ecology & Evolution* **1**, 1–13 (2017).

[2] Villoutreix, R. *et al.* Large-scale mutation in the evolution of a gene complex for cryptic coloration. *Science* **369**, 460–466 (2020).

[3] Nosil, P. *et al.* Genomic consequences of multiple speciation processes in a stick insect. *Proc. R. Soc. B* rspb20120813 (2012).

[4] Hey, J. & Nielsen, R. Multilocus methods for estimating population sizes, migration rates and divergence time, with applications to the divergence of drosophila pseudoobscura and d. persimilis. *Genetics* **167**, 747–760 (2004).

[5] Gutenkunst, R. N., Hernandez, R. D., Williamson, S. H. & Bustamante, C. D. Inferring the joint demographic history of multiple populations from multidimensional SNP frequency data. *PLoS Genetics* **5**, e1000695 (2009).

[6] Nosil, P. *et al.* Ecology shapes epistasis in a genotype–phenotype–fitness map for stick insect colour. *Nature Ecology & Evolution* **4**, 1673–1684 (2020).

[7] Li, H. & Durbin, R. Fast and accurate short read alignment with burrows–wheeler transform. *Bioinformatics* **25**, 1754–1760 (2009).

[8] Li, H. *et al.* The Sequence Alignment/Map format and SAMtools. *Bioinformatics* **25**, 2078–2079 (2009).

[9] Li, H. A statistical framework for snp calling, mutation discovery, association mapping and population genetical parameter estimation from sequencing data. *Bioinformatics* **27**, 2987–2993 (2011).

Supplementary Tables

Supplementary Table 1: Summary of Bayesian model comparison by deviance information criterion (DIC) for habitat and sexual isolation. Penalty denotes the effective number of parameters (a penalty for model complexity). DIC is the sum of the mean deviance (a measure of model fit) and the penalty. The best model, that is the model with the lowest DIC, is highlighted for each component of isolation. All models, including the null models, include an overall intercept and species random effects to account for the same species showing up in multiple comparisons. Source data are provided as a Source Data file.

Type	Model	Mean deviance	Penalty	DIC
Habitat isolation	**Genetics + Taxon + Interaction**	**-46.82**	**9.735**	**-37.08**
	Genetics + Taxon	-39.32	8.249	-31.08
	Genetics	-35.79	7.178	-28.61
	Taxon	-40.30	7.262	-33.03
	Null	-32.20	6.083	-26.12
Sexual isolation	Genetics + Taxon + Interaction	60.51	6.265	66.78
	Genetics + Taxon	59.66	5.229	64.89
	**Genetics**	**58.73**	**4.260**	**62.99**
	Taxon	60.51	4.295	64.81
	Null	61.09	3.543	64.63

Supplementary Table 2: Bayesian parameter estimates for the effects of genetic distance and same versus different species comparison on habitat and sexual isolation based on the full Bayesian linear mixed model that includes these terms and their interaction. This was the best model for habitat isolation; a simpler model with only genetic distance was preferred for sexual isolation. The posterior median point estimate and 2.5th and 97.5th percentiles are given, thus defining the 95% equal-tail probability interval. Only the interaction term for habitat isolation is credibly different from 0. Source data are provided as a Source Data file.

Type	Parameter	Median	2.5th %	97.5th %
Habitat isolation	Genetics	-0.074	-0.18	0.028
	Taxon	-0.043	-0.28	0.19
	Interaction	0.27	0.074	0.46
Sexual isolation	Genetics	0.26	-0.26	0.80
	Taxon	-0.40	-2.16	1.51
	Interaction	0.35	-1.08	1.67

Supplementary Table 3: Divergence time estimates between taxon pairs from a time-calibrated phylogenetic tree. Bayesian point estimates of divergence times are given in millions of years ago (MYA) based on [1]. Pair numbers correspond to those in Supplementary Data 1. Genome-average estimates of F_{ST} from the GBS data are given for comparison.

Pair	Species 1	Species 2	Populations	F_{ST}	Time (MYA)
12	*T. podura*	*T. podura*	BSC X DZRA	0.26	2.49
13	*T. podura*	*T. podura*	BMTC X DZRA	0.1	1.89
15	*T. poppensis*	*T. poppensis*	TBARNF X FROKF13	0.47	1.36
21	*T. chumash*	*T. chumash*	GR6.03Q X HF5Q (HF4)	0.59	5.31
23	*T. chumash*	*T. chumash*	HF6Q X GR10.43Q	0.68	5.31
26	*T. chumash*	*T. chumash*	GR8.06Q X 243BSC	0.72	7.12
33	*T. chumash*	*T. podura*	PFC X PFA	0.89	18.34
35	*T. californicum*	*T. poppensis*	LPQ X LPF	0.83	10.19
36	*T. californicum*	*T. poppensis*	SM1M X SM1RW	0.83	10.19
37	*T. californicum*	*T. poppensis*	SM1Q X SM2RW	0.82	10.19
38	*T. knulli*	*T. landelsnsis*	BCERW X BCHCM	0.73	10.19
39	*T. knulli*	*T. petita*	BCWPC 101SSC	0.52	6.77
40	*T. bartmani*	*T. podura*	JLX SRHWY	0.88	8.36
41	*T. bartmani*	*T. podura*	BMCG3WF X BMCG3Q	0.7	8.36

Supplementary Table 4: Summary of fit of alternative demographic models for intra-specific populations pairs with $\delta a \delta i$. Pair numbers correspond to those in Supplementary Data 1. Log likelihoods (L) and Aikaike Information Criteria (AIC) are reported for each of three demographic models: strict isolation (SI), isolation with migration (IM), and secondary contact (SC). NA denotes a single case where the numerical optimization procedure failed to converge. The AIC for the best model (lowest AIC) is indicated with bold font.

Pair	Species	Populations	L(SI)	L(IM)	L(SC)	AIC_{SI}	AIC_{IM}	AIC_{SC}
12	*T. podura*	BSC X DZRA	-334.80	-201.50	-197.96	679.60	417.00	**411.92**
13	*T. podura*	BMTC X DZRA	-541.78	-388.21	-389.16	1093.57	**790.41**	794.32
15	*T. poppensis*	TBARNF X FROKF13	-4455.43	-2333.64	-2258.50	8920.85	4681.27	**4533.01**
17	*T. knulli*	BCTURNP X BCTURNC	-4853.48	-4963.21	-3254.00	9716.96	9940.43	**6523.99**
18	*T. curi*	CRC X CRA	-2971.05	-2949.93	NA	5952.09	**5913.85**	NA
19	*T. curi*	CRM X CRQ	-372.48	-372.68	-369.71	**754.96**	759.36	755.41
21	*T. chumash*	GR6.03Q X HF5Q	-1283.81	-936.39	-569.89	2577.62	1886.77	**1155.78**
23	*T. chumash*	HF6Q X GR10.43Q	-1337.15	-719.79	-595.59	2684.30	1453.57	**1207.18**
26	*T. chumash*	GR8.06Q X 243BSC	-1959.86	-1072.38	-609.67	3929.72	2158.75	**1235.34**
31	*T. chumash*	BALDQ X BALDC	-318.21	-319.84	-318.85	**646.42**	653.68	653.69

Supplementary Figures

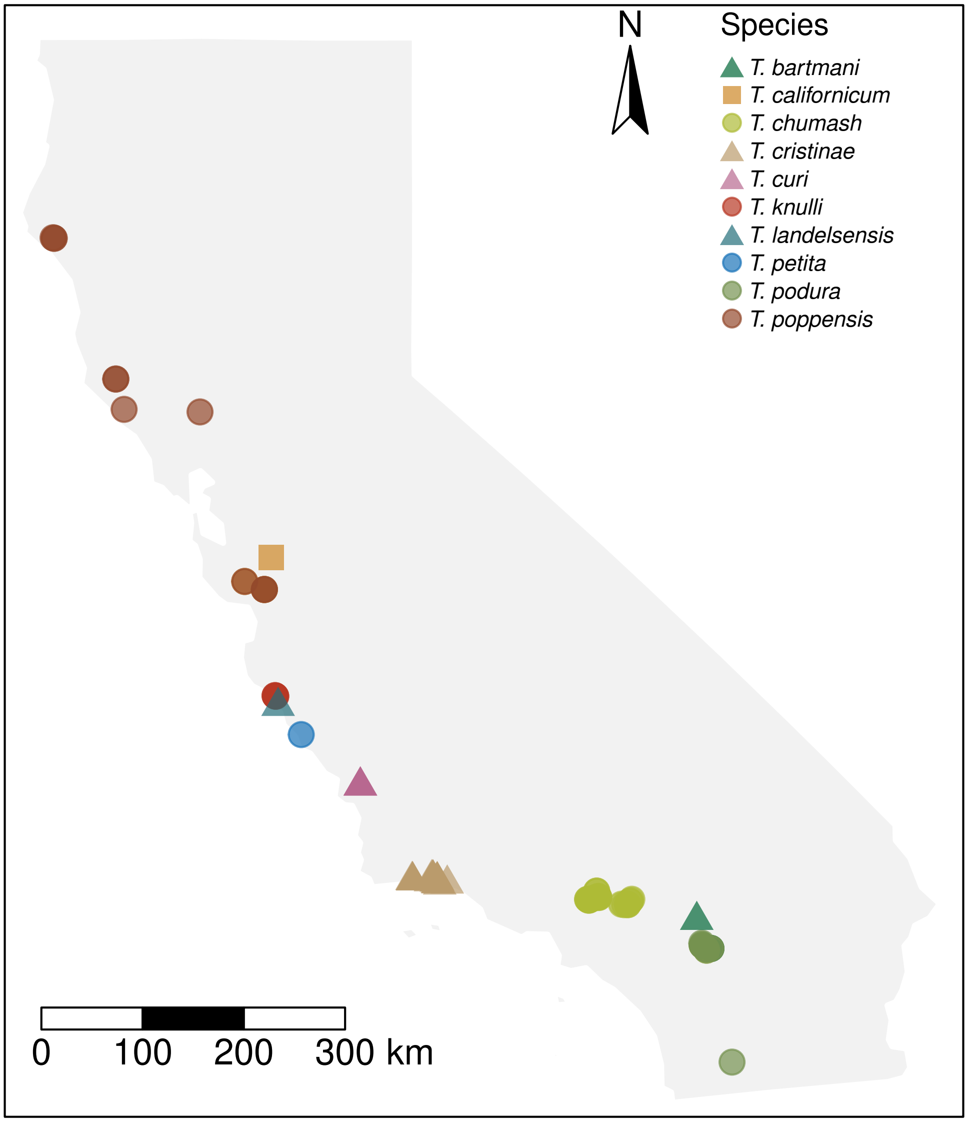

Supplementary Fig. 1: Map depicting the *Timema* species analyzed in this study. Each colored symbol denotes a population with the color and shape of the symbol indicating the species. The outline of California, USA is shown for reference. Population names and coordinates are given in Supplementary Data 2. Source data are provided as a Source Data file.

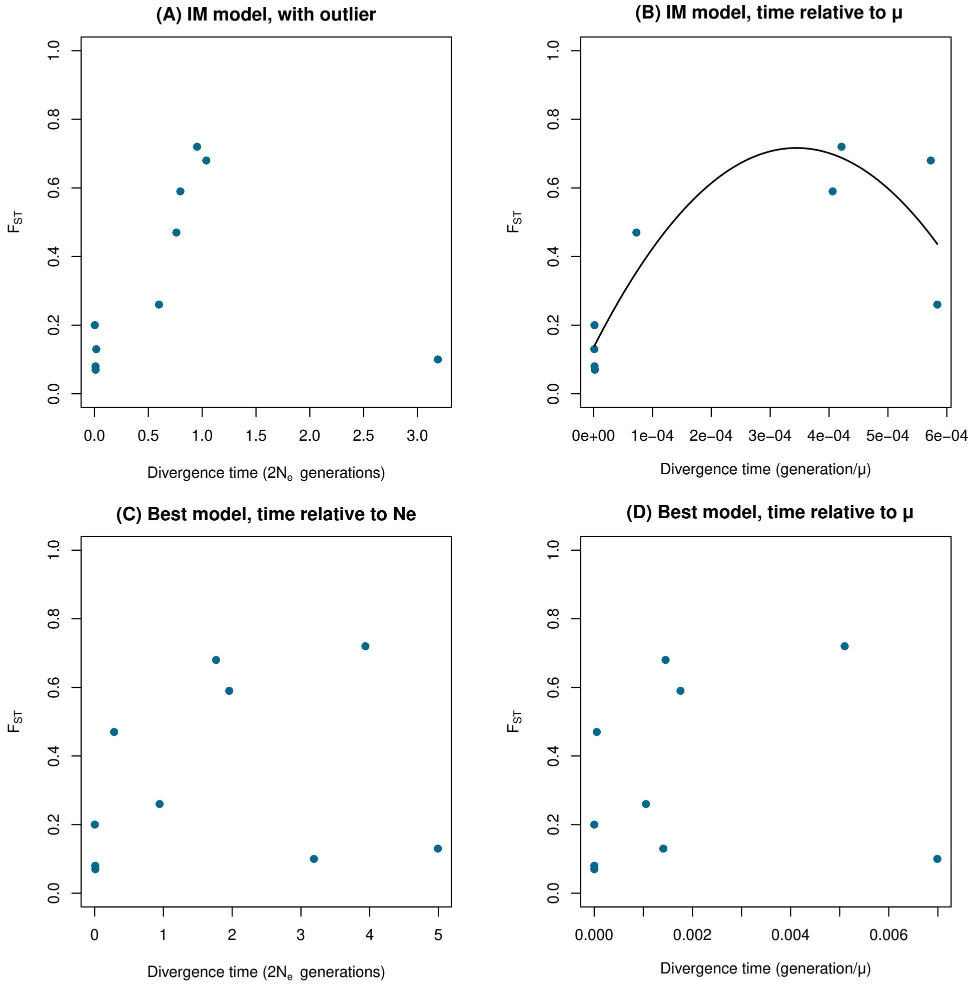

Supplementary Fig. 2: Relationship between divergence time and genome-wide F_{ST} estimated from genotyping-by-sequencing data (GBS) under different conditions. Panel (A) shows F_{ST} as a function of divergence time estimates (in units of 2 N_e generations) from a isolation-with-migration (IM) demographic model with N = 10 population pairs. This plot includes the outlier (divergence time > 3) that was not shown in Fig. 7B. Panel (B) shows F_{ST} as a function of divergence time estimates relative to mutation (μ) and thus removes the effect of variation in effective population size (N_e). This plot excludes the outlier population pair (N = 9 population pairs). The best fit line from polynomial regression is shown (model r^2 = 0.79, P = 0.0098). Panels (C) and (D) shows F_{ST} as a function of divergence time estimates under the best demographic model for each population pair with time in units of $2N_e$ generations (C) or relative to mutation (D) for all N = 10 population pairs. No outliers were removed and no lines are included as the models did not explain a statistically significant proportion of the variation in F_{ST}. Source data are provided as a Source Data file.

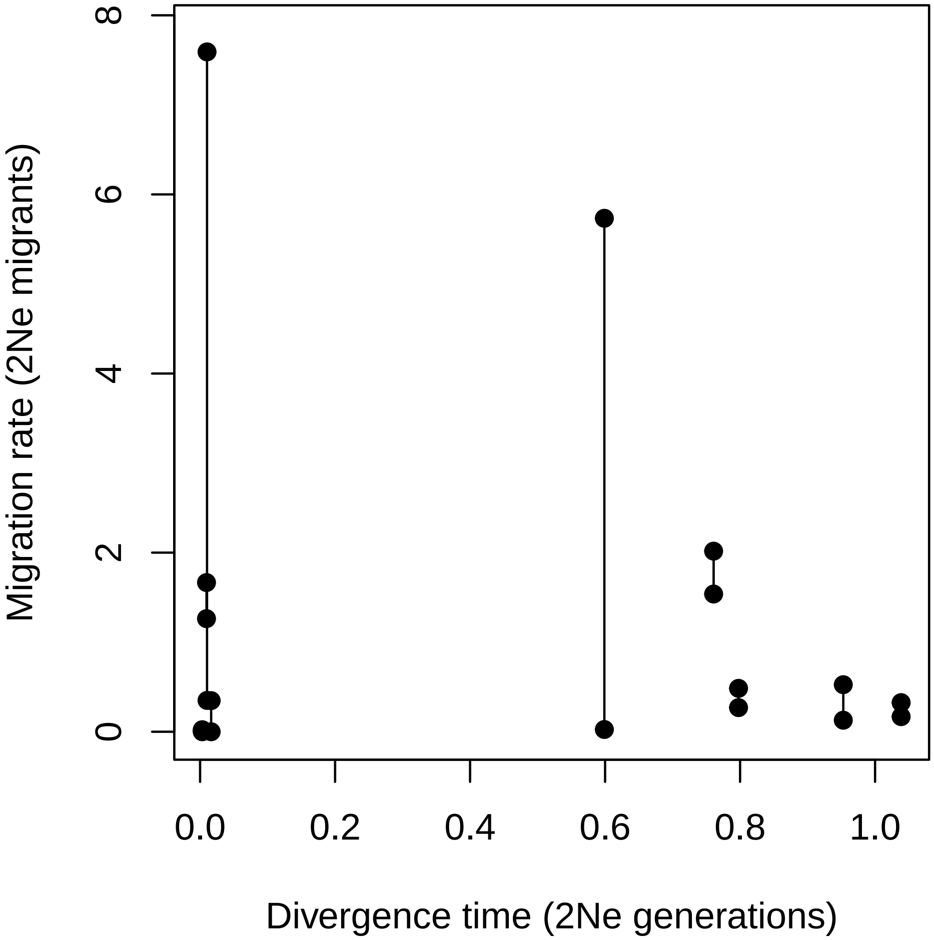

Supplementary Fig. 3: Estimates of gene flow between population pairs from the demographic isolation-with-migration model. Gene flow is shown relative to divergence time estimates (in units of $2N_e$ generations). Each point denotes a gene flow estimate for one of the $N = 9$ population pairs in units of $2N_e$ migrants per generation. Asymmetric gene flow is allowed, so two gene flow estimates are provided per population pair. This are connected by a vertical line. Source data are provided as a Source Data file.
