# Supplementary material for: Divergent dynamics of sexual and habitat isolation at the transition between stick insect populations and species
Source: Nat Commun. 2024 Mar 13;15:2273. doi: 10.1038/s41467-024-46294-9 (PMC10937975; doi:10.1038/s41467-024-46294-9)
Supplement: Supplementary file 3 — Description of Additional Supplementary Files [file 41467_2024_46294_MOESM3_ESM.pdf]

Supplementary Data 1: Characteristics of the 42 population pairs of *Timema*. For geography, A = allopatric, P = parapatric (i.e., sympatric/parapatric). % pick 1 refers to proportion of individuals from population 1 choosing host 1 in the feeding trial and % pick 2 refers to proportion of individuals from population 2 choosing host 1 in the feeding trial. Sexual isolation (SI) is the index of sexual isolation described in the manuscript. NA denotes cases where SI was not computed because of missing data (either no mating trials or no mating trials of a given type).

Supplementary Data 2: Summary of locations of all populations analyzed in this study. These are displayed graphically in Supplementary Fig. 1. Longitude and latitude are given in decimal degrees.

Supplementary Data 3: Sample sizes from the 42 population pairs of *Timema* examined. Pair numbers correspond to those in Supplementary Data 1. HP1 = number of feeding trials for population 1. HP2 = number of feeding trials for population 2. Tested columns refer to number of mating pairs tested in the no-choice mating trails (F1M1 = female from population 1, male from population 1; F1M2 = female from population 1, male from population 2; F2M1 = female from population 2, male from population 1; F2M2 = female from population 2, male from population 2). Copulated columns refer to number of pairs copulating, of those tested. DNA1 and DNA2 give the number of nuclear DNA sequences obtained for populations 1 and 2.

Supplementary Data 4: Summary of genetic distances for population pairs of *Timema*. Pair numbers correspond to those in Supplementary Data 1. nucDNA are mean patristic distances from the nuclear sequence data. GBS is the estimate of F_{ST} for the genomic (GBS) data. GBS populations denotes either the pairs of populations used to estimate F_{ST} or NA if F_{ST} was inferred based on the relationship between GBS-based F_{ST} and nucDNA patristic distance.

Supplementary Data 5: Demographic parameter estimates from dadi. Pair numbers correspond to those in Supplementary Data 1. The parameters are Theta = 4 Ne mu L (here Ne is the ancestral, reference effective population size), the population split time T_1 (in units of 2Ne generations), the time of secondary contact T_2 (also in units of 2Ne generations), and the asymmetric migration rates M_12 and M_21 (both in units of 2Ne migrants per generation) . Estimates are reported for each of three demographic models: strict isolation (SI), isolation with migration (IM), and secondary contact (SC). NA denotes a single case where the numerical optimization procedure failed to converge as well as parameters that are not estimated within subsets of models. The model with the lowest AIC is denoted with an asterisk.
