# Supplementary material for: Divergent dynamics of sexual and habitat isolation at the transition between stick insect populations and species
Source: Nat Commun. 2024 Mar 13;15:2273. doi: 10.1038/s41467-024-46294-9 (PMC10937975; doi:10.1038/s41467-024-46294-9)
Supplement: Supplementary file 5 — Reporting Summary [file 41467_2024_46294_MOESM5_ESM.pdf]

Corresponding author(s): Daniel J. Funk
Last updated by author(s): Jan 23, 2024

Reporting Summary

Nature Portfolio wishes to improve the reproducibility of the work that we publish. This form provides structure for consistency and transparency in reporting. For further information on Nature Portfolio policies, see our Editorial Policies and the Editorial Policy Checklist.

Statistics

For all statistical analyses, confirm that the following items are present in the figure legend, table legend, main text, or Methods section.

n/a	Confirmed	
☐	☒	The exact sample size (*n*) for each experimental group/condition, given as a discrete number and unit of measurement
☐	☒	A statement on whether measurements were taken from distinct samples or whether the same sample was measured repeatedly
☐	☒	The statistical test(s) used AND whether they are one- or two-sided<br>*Only common tests should be described solely by name; describe more complex techniques in the Methods section.*
☐	☒	A description of all covariates tested
☐	☒	A description of any assumptions or corrections, such as tests of normality and adjustment for multiple comparisons
☐	☒	A full description of the statistical parameters including central tendency (e.g. means) or other basic estimates (e.g. regression coefficient) AND variation (e.g. standard deviation) or associated estimates of uncertainty (e.g. confidence intervals)
☐	☒	For null hypothesis testing, the test statistic (e.g. *F*, *t*, *r*) with confidence intervals, effect sizes, degrees of freedom and *P* value noted<br>*Give P values as exact values whenever suitable.*
☐	☒	For Bayesian analysis, information on the choice of priors and Markov chain Monte Carlo settings
☐	☒	For hierarchical and complex designs, identification of the appropriate level for tests and full reporting of outcomes
☐	☒	Estimates of effect sizes (e.g. Cohen's *d*, Pearson's *r*), indicating how they were calculated

Our web collection on statistics for biologists contains articles on many of the points above.

Software and code

Policy information about availability of computer code

Data collection	No software was used for data collection.
Data analysis	The following software packages were used for data analysis:<br>PhyML version 3.0<br>R version 4.2.2<br>JAGS version 4.13<br>coda (R package) version 0.19.4<br>strucchange (R package) version 1.5.3<br>dadi (no version number available)<br>python3<br>Custom R, Bash, Python and Perl scripts available from GitHub https://github.com/zgompert/TimemaRI and Zenodo https://doi.org/10.5281/zenodo.8312010

For manuscripts utilizing custom algorithms or software that are central to the research but not yet described in published literature, software must be made available to editors and reviewers. We strongly encourage code deposition in a community repository (e.g. GitHub). See the Nature Portfolio guidelines for submitting code & software for further information.

Data

Policy information about availability of data

All manuscripts must include a data availability statement. This statement should provide the following information, where applicable:

- Accession codes, unique identifiers, or web links for publicly available datasets
- A description of any restrictions on data availability
- For clinical datasets or third party data, please ensure that the statement adheres to our policy

The reanalyzed DNA sequence data are available from the NCBI SRA database under accession PRJNA356405 https://www.ncbi.nlm.nih.gov/bioproject/356405). All other data are available from Dryad (doi:10.5061/dryad.q573n5tpk) and as a Source Data File associated with this manuscript.

Research involving human participants, their data, or biological material

Policy information about studies with human participants or human data. See also policy information about sex, gender (identity/presentation), and sexual orientation and race, ethnicity and racism.

Reporting on sex and gender	Not applicable, this research does not involve human participants or their associated data.
Reporting on race, ethnicity, or other socially relevant groupings	Not applicable, this research does not involve human participants or their associated data.
Population characteristics	Not applicable, this research does not involve human participants or their associated data.
Recruitment	Not applicable, this research does not involve human participants or their associated data.
Ethics oversight	Not applicable, this research does not involve human participants or their associated data.

Note that full information on the approval of the study protocol must also be provided in the manuscript.

Field-specific reporting

Please select the one below that is the best fit for your research. If you are not sure, read the appropriate sections before making your selection.

☐ Life sciences ☐ Behavioural & social sciences ☒ Ecological, evolutionary & environmental sciences

For a reference copy of the document with all sections, see nature.com/documents/nr-reporting-summary-flat.pdf

Ecological, evolutionary & environmental sciences study design

All studies must disclose on these points even when the disclosure is negative.

Study description	We used thousands of host-preference and mating trials to study habitat and sexual isolation among N = 42 pairs of Timema stick insects. These population pairs served as the unit of replication for core analyses (which accounted for the pairwise nature of the data). Host-preference trials presented individual stick insects with cuttings from two host-plant species. Sexual isolation trials involved pairs of adult male and female stick insects from either the same or different taxa (this was the treatment, same versus different taxon).
Research sample	The research sample was 42 population pairs of Timema stick insects. The number of pairs was based on what was logistically possible. Beyond that, population pairs were chosen to maximize within versus between species contrasts along with geographic contrasts. Stick insects from a given population are meant to be representative of that population. Sexually mature adults were used for sexual isolation trials.
Sampling strategy	As in past work, we define a population as all of the Timema stick insects collected within a homogeneous patch of a single host species at a given geographic locality.
Data collection	All specimens used in this study were collected using sweep nets. This involves beating the branches of host plants with a stick and capturing (with a sweep net) the stick insects that are shaken free. Data for analyses were collected in the field by direct observation of the host-preference and mating trials. Data were collected by or under the supervision of P. Nosil.
Timing and spatial scale	Data were collected during the spring, as this is the period of time Timema stick insects are alive and active (specific dates vary by site based on the species and climatic conditions). Specimens were collected from individual stick insect populations, defined as the Timema stick insects within a homogeneous patch of a single host. Populations were collected across California, as this is the combined range of the species studied. All spatial details are provided in the manuscript.
Data exclusions	In general, no data were excluded. The one exception is that one pair was excluded from some plots involving divergence time

Data exclusions	because it's divergence time was several times larger than all other pairs. This is explained in the manuscript and the plots with the pair included are provided in the Supplementary Information.
Reproducibility	No repeated measures were taken, but the study involves substantial replication within and across taxon pairs.
Randomization	Organisms were allocated to groups based on their taxonomic classification and the host plant and geographic location from which they were sampled. In other words, nature allocated the organisms to groups, not the researchers.
Blinding	Blinding was not possible during data collection as the species of each stick insect is evident and assays were conducted in the field so geographic information was necessarily known. Host-preference and mating trials were scored without direct reference to the population involved.

Did the study involve field work? ☒ Yes ☐ No

Field work, collection and transport

Field conditions	Field work was conducted during the day in the spring and summer; this time period is mostly warm and sunny in the geographic regions occupied by the Timema stick insects.
Location	Samples were collected from 42 population pairs across California. Details on sampling locations are provided in the Supplementary Data 2 in the manuscript.
Access & import/export	Permits were not required for invertebrate sampling. Access to sites was by car and then by foot.
Disturbance	The study resulted in minimal disturbance.

Reporting for specific materials, systems and methods

We require information from authors about some types of materials, experimental systems and methods used in many studies. Here, indicate whether each material, system or method listed is relevant to your study. If you are not sure if a list item applies to your research, read the appropriate section before selecting a response.

Materials & experimental systems

n/a	Involved in the study	
☒	☐	Antibodies
☒	☐	Eukaryotic cell lines
☒	☐	Palaeontology and archaeology
☐	☒	Animals and other organisms
☒	☐	Clinical data
☒	☐	Dual use research of concern
☒	☐	Plants

Methods

n/a	Involved in the study	
☒	☐	ChIP-seq
☒	☐	Flow cytometry
☒	☐	MRI-based neuroimaging

Animals and other research organisms

Policy information about studies involving animals; ARRIVE guidelines recommended for reporting animal research, and Sex and Gender in Research

Laboratory animals	No laboratory animals were used in this study.
Wild animals	This work involved collecting and assaying Timema stick insects. All specimens used in this study were collected using sweep nets. This involves beating the branches of host plants with a stick and capturing (with a sweep net) the stick insects that are shaken free. Mate preference trials involved virgin adults; host preference trials included a range of ages of stick insects (this is a univoltine species and thus all <1 year old). Stick insects were killed by submersion in ethanol after the experiments. This was done to preserve tissue for DNA and to minimize effects on the actual wild populations.
Reporting on sex	Findings are not sex-specific. Host preference trials resulted in population-level inferences and the mate preference trials necessarily involved one male and one female stick insect each.
Field-collected samples	For host preference trials, each evening, individual stick insects were placed in a 500 milliliter plastic cup with ~15 cm cuttings of two different host-plant species. The cups were covered with mosquito mesh and left overnight. In the morning we scored which of the two plant species the stick insect was resting upon. Trials were conducted in the field under ambient light and temperature conditions, or under similar conditions in the laboratory. For mating preference trials, one adult male and one adult female were placed in a standard 10 cm petri dish. Each pair was observed for one hour and scored as having mated or not (Timema have an extended copulation period that is unambiguous and easy to observe).

Ethics oversight	This work involved insects and thus no ethics approval was required.

Note that full information on the approval of the study protocol must also be provided in the manuscript.

Plants

Seed stocks	Not applicable, we did not study plants.
Novel plant genotypes	Not applicable, we did not study plants.
Authentication	Not applicable, we did not study plants.
